# ^11^C-PET imaging reveals transport dynamics and sectorial plasticity of oak phloem after girdling

**DOI:** 10.3389/fpls.2013.00200

**Published:** 2013-06-18

**Authors:** Veerle De Schepper, Jonas Bühler, Michael Thorpe, Gerhard Roeb, Gregor Huber, Dagmar van Dusschoten, Siegfried Jahnke, Kathy Steppe

**Affiliations:** ^1^Laboratory of Plant Ecology, Department of Applied Ecology and Environmental Biology, Faculty of Bioscience Engineering, Ghent UniversityGhent, Belgium; ^2^IBG-2: Plant Sciences, Forschungszentrum JülichJülich, Germany; ^3^Research School of Biology, Australian National UniversityCanberra, ACT, Australia; ^4^High Resolution Plant Phenomics Centre, CSIRO Plant IndustryCanberra, ACT, Australia

**Keywords:** assimilates, girdle, *Quercus robur* L., tracer model, translocation, wounding

## Abstract

Carbon transport processes in plants can be followed non-invasively by repeated application of the short-lived positron-emitting radioisotope ^11^C, a technique which has rarely been used with trees. Recently, positron emission tomography (PET) allowing 3D visualization has been adapted for use with plants. To investigate the effects of stem girdling on the flow of assimilates, leaves on first order branches of two-year-old oak (*Quercus robur* L.) trees were labeled with ^11^C by supplying ^11^CO_2_-gas to a leaf cuvette. Magnetic resonance imaging gave an indication of the plant structure, while PET registered the tracer flow in a stem region downstream from the labeled branches. After repeated pulse labeling, phloem translocation was shown to be sectorial in the stem: leaf orthostichy determined the position of the phloem sieve tubes containing labeled ^11^C. The observed pathway remained unchanged for days. Tracer time-series derived from each pulse and analysed with a mechanistic model showed for two adjacent heights in the stem a similar velocity but different loss of recent assimilates. With either complete or partial girdling of bark within the monitored region, transport immediately stopped and then resumed in a new location in the stem cross-section, demonstrating the plasticity of sectoriality. One day after partial girdling, the loss of tracer along the interrupted transport pathway increased, while the velocity was enhanced in a non-girdled sector for several days. These findings suggest that lateral sugar transport was enhanced after wounding by a change in the lateral sugar transport path and the axial transport resumed with the development of new conductive tissue.

## Introduction

Tree physiology studies have often used the radio-active carbon isotope ^14^C (e.g., Lockhart et al., 2003; Sloan and Jacobs, 2008; Bonhomme et al., 2010). But the short-lived tracer ^11^C has a high scientific potential, since tracer dynamics can be observed non-invasively in a plant for an unlimited period, provided the tracer is available (Minchin and Thorpe, 2003). Yet it has been used in very few tree studies (Lang and Minchin, 1986; Jahnke et al., 1998; McQueen et al., 2005). Due to its short half-life time of 20.4 min, ^11^C must be produced close to the experimental site. Hence, the reason for the scarcity of ^11^C experiments on living trees is the necessity of a nearby cyclotron. The short half-life time of ^11^C restricts the time-scales of processes that can be studied, but changes in those processes can be followed in consecutive tests, since there is no build-up of the tracer with repeated labeling. The applicability of ^11^C tracer technique in plant research is illustrated by the wide variety of studies that have used it in plant physiology (e.g., Freckman et al., 1991; Jahnke et al., 1998; Gould et al., 2004; McQueen et al., 2005; Thorpe et al., 2010).

The radionuclide ^11^C is mostly administered to plant leaves as ^11^CO_2_ which is fixed by photosynthesis. Upon decay, ^11^C releases a positron (β^+^) which is subsequently annihilated to give two γ-rays (511 KeV photons) after collision with an electron. The path from decay to annihilation has a maximum of about 4 mm in water, limiting the spatial resolution if detecting γ photons. The photons can be observed singly or by coincidence, using scintillation detectors. If singly, the regions of interest are defined by suitable radiation shielding, giving ^11^C tracer time-series for the radioactivity in each region (Minchin and Thorpe, 2003). Recently, a positron emission tomography (PET) system was developed for plants at Forschungszentrum Jülich, the Plant Tomographic Imaging System (PlanTIS) (Beer et al., 2010). In contrast to single-photon detectors, this system counts events only when two annihilation γ-rays coincide in time. Due to this specific characteristic, PlanTIS can provide three-dimensional (3D) images of ^11^C labeled assimilates without the need for radiation shielding (Jahnke et al., 2009). Additionally, a much more flexible examination of tracer flow is possible because ^11^C tracer time-series for any region of interest can be extracted from these images after the experiment. The tracer profiles can be analysed with a semi-mechanistic compartmental model describing long-distance tracer-transport (Bühler et al., 2011). Parameter optimization by fitting modeled data to experimental data gives physiological information on translocation velocity and tracer exchange between the pathway and adjacent tissues.

We observed that PlanTIS, with its expected spatial resolution of about 2 mm, could portray useful three-dimensional images of ^11^C distribution during translocation in the stem of young oak trees with a stem diameter of only 13 mm. This capability was needed to study the influence of girdling on the assimilate flow in the stem of oak trees, which was the main objective of this work. More specifically, this study aimed to reveal the effect of girdling on the tracer's pathway within the stem cross-section, on its axial velocity, and on its loss (unloading) into tissues adjacent to its pathway. 3D images obtained with PlantTIS gave the position of ^11^C in the plane of the stem cross-section, and model analyses of temporal ^11^C tracer profiles provided information about the tracer's velocity and loss along the transport pathway. In order to illustrate the location of the tracer pathway within the stem, the anatomy of the oak stem was obtained by magnetic resonance imaging (MRI)

## Materials and methods

### Plant material and experimental treatments

**Figure 1:**
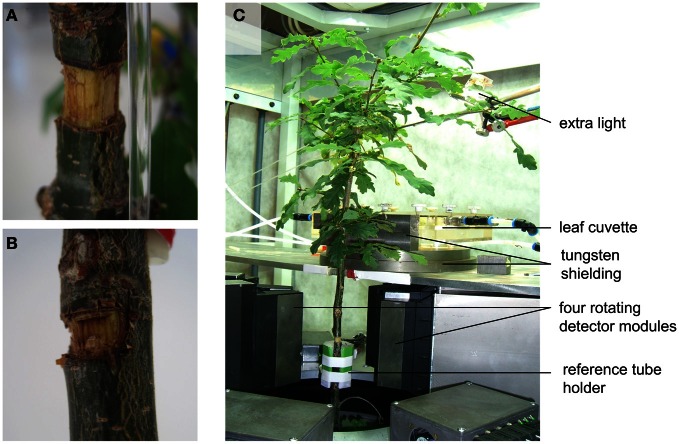
**The experimental system.** Photographs of **(A)** complete girdling, **(B)** partial girdling, and **(C)** experimental setup of a potted oak tree in the PET system “PlanTIS” at the Forschungszentrum Jülich.

Measurements were conducted on two-year-old oak trees (*Quercus robur* L.). On 15 March, these trees were planted in 10-liter containers filled with a potting mixture (LP502D, Peltracom nv, Belgium) and fertilized with a slow releasing NPK plus magnesium mix (Basacote Plus 6M, COMP0, Benelux). The trees were irrigated every 2–3 days to ensure the potting mixture remained well-watered. At the time of measurements in October, the trees had a stem diameter of approximately 1.3 cm and a height of approximately 110 cm (Figure 1). Two types of girdling were performed. In one case, a 1 cm strip of bark was removed around the complete outer circumference of the oak stem, henceforth referred to as the complete girdling treatment (Figure 1A). In a second case, a strip of bark was removed around half of the stem outer circumference, henceforth referred as partial girdling (Figure 1B). At planting, the lower branches of these trees were removed so no branches were present below the girdle.

### Pet data collection

**Figure 2:**
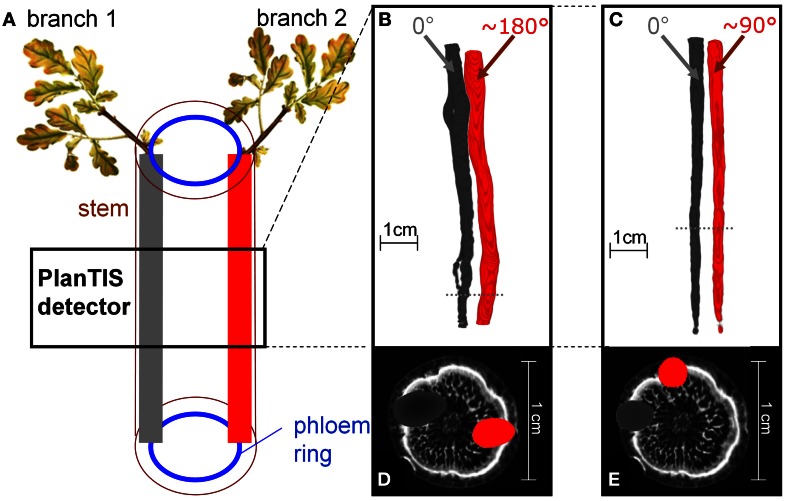
**The location of ^11^C tracer flow in the stem of an oak tree depended on the position of the branch which was labeled. (A)** Schematic presentation of how the tree's structure related to the PET images from labeling two branches. **(B,C)** PET 3D-images from two trees, showing ^11^C distribution along the stem from two different branches. The branches, labeled in consecutive experiments (gray and red), were inserted at angles **(B)** ~180° and **(C)** ~90°. Dotted lines indicate the positions of the cross-sections in **(D)** and **(E)**. **(D,E)** The respective cross-sections of the PET images (gray, red) overlaid with MRI (gray scale) water content images not of the same, but a similar oak tree for illustration.

PlanTIS is a high-resolution PET scanner which allows *in vivo* visualization of positron emitters like ^11^C in plants by creating 3D-images (Jahnke et al., 2009). Its detectors, front-end electronics and data acquisition architecture are based on the ClearPET™ system (Streun et al., 2006). Two groups of four detector modules stand face-to-face and rotate around a vertical axis so plants can be placed in their natural upright position. More details about this PET system can be found in Beer et al. (2010). The oak trees were placed in the PET system (Figure 1C) at least 1 day before the measurements to allow acclimatization of physiological plant processes, especially photosynthesis and translocation. The cylindrical field of view (FOV) contained an 11-cm-long region of the main stem and three reference sources (glass capillaries filled with liquid [^11^C]NaHCO_3_) placed around the stem for co-registration of images from repeated runs. By supplying ^11^CO_2_-gas to a leaf cuvette connected to a closed gas-exchange system, approximately 85 cm^2^ of leaves on first order branches inserted at nodes between 30 and 40 cm above the FOV became radioactively labeled as schematically illustrated in Figure 2A. The signal of the ^11^C tracer was measured every 5 min for about 2.5 h after labeling. One branch was pulse-labeled at 10:30 in the morning, the other at 14:00 in the afternoon when tracer from the first pulse had become negligible due to decay. During the daytime (from 7:30 till 19:30), the plants in PlanTIS were subjected to PAR ranging from 200 to 480 μmol m^−2^ s^−1^ (approximately 350 μmol m^−2^ s^−1^ at the labeled leaf). The imposed temperature and relative humidity fluctuated according to room conditions: night-day temperatures were 18–24°C and night-day relative humidity was 30–60%.

### Pet data analyses

**Figure 3:**
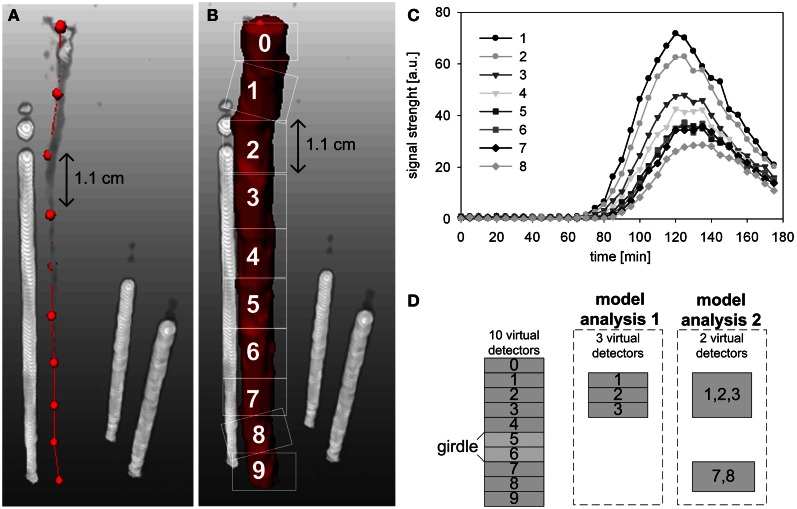
**Time series were generated for analysis from PET images of tracer in the stem of an oak tree by using ten virtual detectors. (A)** The centers of the virtual detectors are indicated by red dots. **(B)** Regions of signal detection within the PET image are indicated by white squares. The white rods show the three reference sources (glass capillaries filled with liquid [^11^C]NaHCO_3_) needed for co-registration of images from repeated runs. **(C)** Temporal ^11^C tracer profiles measured at the virtual detectors 1–8, respectively. **(D)** Schematic presentation of the virtual detectors that were selected for analyses of phloem transport above the girdle location (model analysis 1) or spanning the girdle location (model analysis 2).

The PET data were converted by specific image reconstruction-tools to 3D-images (Beer et al., 2010). The reconstructed PET data were additionally converted by an image processing environment (MeVisLab, version 2.1; MeVis Medical Solutions AG, Bremen, Germany) to temporal profiles of ^11^C tracer (Figure 3C), which mimicked the output of ten virtual detectors along the measured stem segment with a thickness of approximately 1.1 cm (Figures 3A,B). Detectors 0 and 9 were omitted from further analyses as they extended beyond the upper and lower edge of the PlanTIS FOV. The conversion was performed in a similar way as in Bühler et al. (2011): the spatial 3D-data were integrated over the FOV of each virtual detector in such a way the ^11^C profiles represent projections of transported ^11^C tracer on a one-dimensional pathway through the stem (Figures 3A,B).

Temporal ^11^C tracer profiles (Figures 3C,D) measured along the stem were analysed with the mechanistic model of Bühler et al. (2011). This model describes tracer transport, exchange and decay within three compartments using only a few transport and exchange parameters and containing no details on spatial scales or anatomy. The three model compartments represent a conduit in which tracer is carried, a transient storage compartment (e.g., parenchyma) exchanging tracer with the local pathway, and a compartment where tracer becomes immobilized. The numerical solution of the partial differential model equations was fitted to the ^11^C tracer profiles (Figures 3C,D), resulting in estimates of tracer transport properties, namely the speed of the radiotracer [mm min^−1^] and the fractional net loss of tracer [% cm^−1^] by steady fixation along the transport pathway (Bühler et al., 2011). The modeled loss is calculated as the amount of tracer fixed in the region of interest relative to the amount of tracer entering this region (of each virtual detector, Figure 3). Hence, the loss is the unloaded tracer at a location, as a fraction of the tracer that has entered that region. The 90% confidence intervals were calculated using a bootstrapping approach (Bühler et al., 2011) to show uncertainty of the estimates.

In order to estimate the transport properties at different locations in the partially girdled oak stem, two model analyses were performed. The first model analysis was based on ^11^C-radiotracer profiles provided by three consecutive virtual detectors above (upstream) the girdle, namely detectors 1, 2, and 3 in Figure 3D. The second analysis was based on two remote detectors: one detector above the girdle comprised of detectors 1, 2, and 3 and one below the girdle comprised of detectors 7 and 8 (Figure 3D). These specific detectors were selected because only they provided data consistent with the model assumption of an uniform system for all data sets.

### MRI image of stem cross-section

PET images of successive ^11^C runs were overlayed on a MRI water-content image of the oak tree used for partial girdling. It should be noted that these MRI images are used here as illustration of vascular stem anatomy and that they were not co-registered with the PET images. The water content images were obtained in a MRI system at Forschungszentrum Jülich (IBG-2) (De Schepper et al., 2012). A 1.5T MRI system was used consisting of a split-coil magnet (Magnex/Agilent, Oxford, UK) and a NMR imaging spectrometer (Varian/Agilent, Alto Palo, USA). Parallel magnetic field gradient-inserts at a separation of 120 mm were used (plate diameter 40 cm, gradient strength up to 800 mT/m). In between these two inserts a part of the oak stem was placed. A small radio-frequency (RF) coil was wound around the stem prior to the measurements, giving a much higher signal to noise ratio compared to a standard whole body RF coil. Images were acquired using (multiple) spin echoes (Haacke et al., 1999). MRI images can serve as a good approximation of the local water amount when the delay (the so-called echo time) between signal excitation and detection is minimized, in this case to 5.4 ms. A slice thickness of 2.5 mm was used with a fixed in-plane resolution (pixel size) of 100 μm. FOV was adapted according to the stem diameter.

## Results

### Vascular architecture

Images of ^11^C distribution in the measured stem segment showed that when different branches above the field of view were labeled, ^11^C flow occurred in distinct regions of the stem cross-section (Figures 2B–E). Figure 2A illustrates schematically the sectorial flow of assimilates in the stem. Figures 2B,C depict PET images from two different oak trees, shown as isosurfaces of ^11^C radioactivity integrated over the time of the experiments. The angle between the insertions of the two labeled branches was approximately 180° (Figures 2B,D) and 90° (Figures 2C,E). In Figure 2B the round gaps in the tracer flow are where a branch had been removed at planting. Cross-sections of the PET images overlaid on the MRI image of a similar oak tree (Figures 2D,E) illustrate that the tracer was located in the phloem region of the stem, which is represented by the peripheral white band in the MRI image (De Schepper et al., 2012). The spatial resolution of PET images in biological tissues is about 2 mm for ^11^C due to the path length of positrons (with a maximum energy of 1 MeV) in tissues. For that reason, the visualized ^11^C flow appears as a cloud around any source (Figures 2D,E).

### Position of carbon flow in the stem before and after girdling

**Figure 4:**
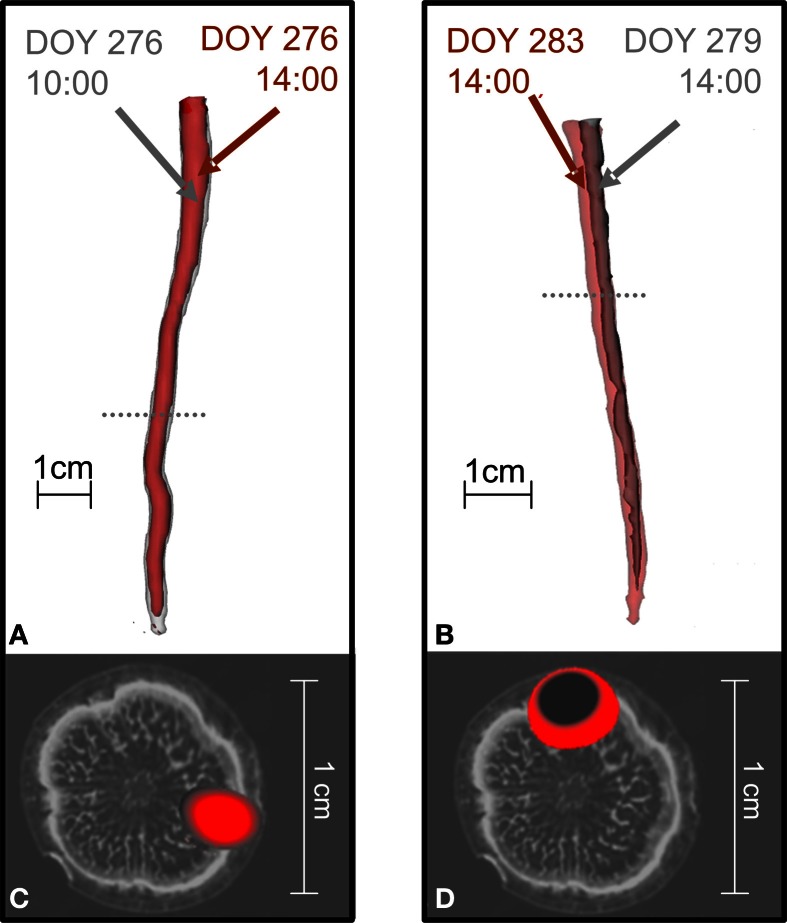
**The position of ^11^C flow in the stems of oak trees did not change when the same branch was repeatedly labeled.** In the tree shown in **(A,C)** a branch was labeled with ^11^CO_2_ at different times on the same day (10:00 red, 14:00 gray) while the tree shown in **(B,D)** was labeled at different days [Day Of the Year (DOY) 279 gray, DOY 283 red]. **(A,B)** Show 3D-PET images of ^11^C distribution along the stems and **(C,D)** cross-sections of the PET images at the positions marked by the dotted lines in **(A,B)** overlaid on an MRI image of a similar oak tree for illustration (cf. Figure 2).

Before treatment, the labeled carbon flow did not change position in time. Figure 4 compares the distribution of radiotracer in oak stems derived from the same branches labeled at different times, showing that the position of the transport paths remained the same. For the two trees presented, the time difference between two depicted tracer profiles of the same branch was 4 h (Figures 4A,C) or 5 days (Figures 4B,D).

**Figure 5:**
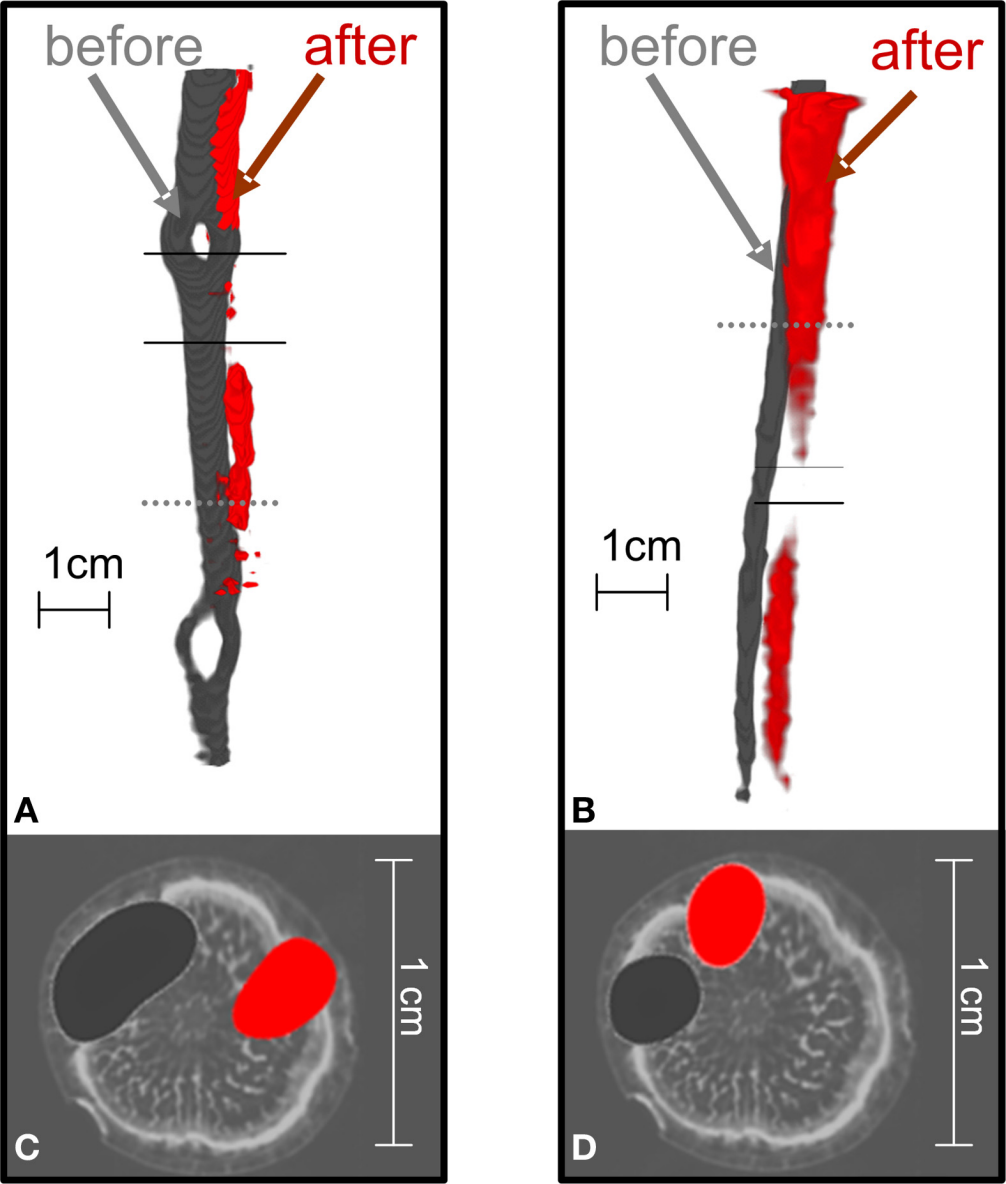
**The position of ^11^C flow pathway in oak stems changed after the complete or partial girdling shown in Figure 1.** 3D-Images of tracer pathways are shown for **(A)** complete girdling, and **(B)** partial girdling. With partial girdling, bark was excised from the labeled sector. The cross-sections in **(C)** and **(D)** (at the dotted lines in **A,B**) are overlaid on MRI images of a similar untreated oak tree for illustration. The radiotracer images were before (dark gray) or after (red) girdling. In **(A)**, the red image was 13 days after complete girdling when new phloem tissue was grown, and in **(B)** 2 days after partial girdling. The two solid lines indicate the girdled region.

When the trees were girdled, the position of radiotracer near the girdle changed from its initial position. Figure 5 visualizes the flow of ^11^C radiotracer before and after complete girdling (Figures 5A,C; cf. Figure 1A) or partial girdling (Figures 1B, 5B,D). The time difference between the two ^11^C labelings of Figures 5A,C (complete girdling experiment) was 13 days while in Figures 5B,D (partial girdling experiment) it was 2 days. In case of partial girdling (Figures 5B,D), the depicted transport route originally passed through the girdled sector. These images of radiotracer distribution show that the path of ^11^C transport in the girdled pathway changed its position to a different sector in the cross-section of the stem at latest 1 day after the (partial) girdling. The ^11^C distribution after girdling (Figures 5A,B, red) appears to be interrupted at the girdle, because the signal density dropped below the image-threshold chosen to eliminate noise from the displayed PET-data. This local decrease in signal density (amount of signal per volume) at the girdle can be attributed to (i) a local increased velocity or/and (ii) a local increased phloem cross-section containing radio-active assimilates. An increased cross-section would indicate that the radio-active assimilates are spread over more phloem vessels/sectors. However, the latter seems unlikely because the phloem cross-section is largely reduced after girdling and wound tissue grows in a non-evenly distributed way at the girdle (De Schepper et al., 2010). Due to the low signal to noise ratio, the actual assimilate pathway at the girdles could not be depicted. Partial girdling did not alter the position of transport routes that did not originally pass the girdle (data not shown).

### Model analysis

**Figure 6:**
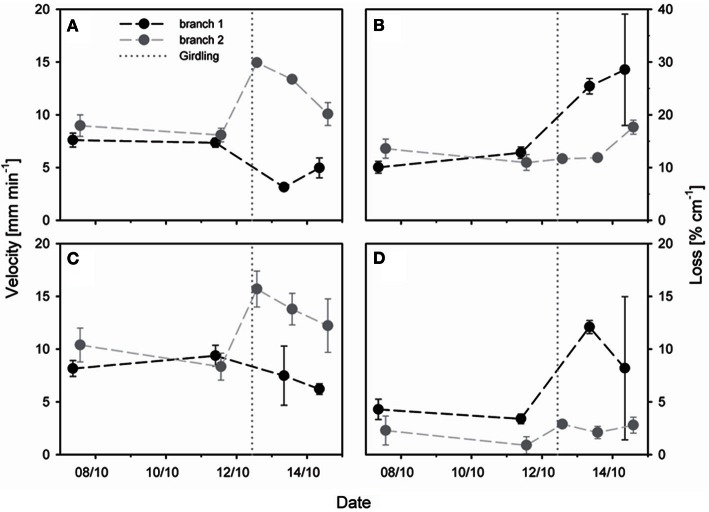
**Effect of partial stem girdling on phloem transport in an oak stem.** Velocity and tracer loss were estimated with a mechanistic tracer model based on temporal ^11^C profiles of **(A,B)** three virtual detectors above (upstream) the girdle (model analysis 1, Figure 3D) or of **(C,D)** two virtual detectors located above and below the girdle (model analysis 2, Figure 3D). Original flow from branch1 was in the girdled sector. The dotted vertical lines represent the time of partial girdling. Error bars indicate 90% confidence intervals. Note that scaling is more sensitive in **(D)** than in **(B)**.

Before girdling, the estimated transport velocity and loss of different oak trees calculated above the prospective girdling zone were very similar: the transport velocity (±standard deviation) was 10.9 ± 4.2 mm min^−1^ (data not shown) before complete girdling and 8.0 ± 0.7 mm min^−1^ before partial girdling (Figure 6A); the loss of radiotracer was 9.2 ± 3.2% cm^−1^ (data not shown) before complete girdling while it was 11.8 ± 1.6% cm^−1^ before partial girdling (Figure 6B). The complete girdled tree was excluded from further model analyses, because only one measurement after girdling was available for this tree (13 days after girdling; cf. Figure 5A).

The effect of girdling on the transport velocity and radiotracer loss was investigated for 3 days after partial girdling. We analysed radiotracer flow emanating from two different branches (Figure 6): for branch 1 the transport route originally passed through the girdled sector and was thus interrupted by girdling, whereas for branch 2 it was outside of the girdled sector and not interrupted. After partial girdling, the transport velocities of both flows (branches 1 and 2) changed: there was a decrease in the flow from branch 1 and an increase in the flow from branch 2, calculated either from three detectors above the girdle (Figure 6A), or from two detectors with one above and one below the girdle (Figure 6C).

The tracer loss along the stem calculated from the three detectors above the girdle (Figure 6B) was much larger than that from the two detectors spanning the girdle (Figure 6D). Initially tracer loss of branch 1 was ~10 % cm^−1^ for the upstream region (Figure 6B) and ~5% cm^−1^ for the entire stem zone (Figure 6D). In both cases the loss increased substantially during 2 days after partial girdling: up to values of ~30% cm^−1^ for the upstream region (Figure 6B) and ~11% cm^−1^ for the entire stem zone (Figure 6D). In contrast, for branch 2 the loss (~12% cm^−1^ for the upstream region and ~3–5% cm^−1^ for the entire stem zone) was much less affected by the partial girdle compared to branch 1 (Figure 6B).

## Discussion

### Assimilate flow in oak stem is sectorial

Young oak trees showed a sectoriality of assimilate flow, with radiotracer distribution in the stem depending on the node of the labeled branch (Figure 2). Similar sectoriality of flow between shoot and roots has been reported earlier in herbaceous species and trees (Stieber and Beringer, 1984; Watson and Casper, 1984; Marshall, 1996; Vuorisalo and Hutchings, 1996; Jahnke et al., 2009). In general, sectoriality arises when the vascular connections between sources and sinks restrict the movement of assimilates in such a way that carbon fixed by a leaf remains primarily within its orthostichy (Fetene et al., 1997; Preston, 1998). Sources preferentially support sinks located above or below the respective insertion node, according to their vascular connections (Taiz and Zeiger, 2002). Therefore, the allocation pattern of assimilates from a source leaf to a particular sink becomes quite predictable from basic information on the geometry of the leaf arrangement on the stem (Marshall, 1996) which is useful for modeling purposes.

### Sectorial plasticity of assimilate translocation

On the days following complete girdling, ^11^C transport in the downward direction was not detected directly above or below the girdle (data not shown), showing that the physical interruption of the phloem pathway had completely disturbed the downward transport of assimilate from that branch. The observation of a new location of assimilate flow 13 days after complete girdling was related to the formation of new phloem tissue spanning the girdle (Figure 5A). It is known for oak that newly formed phloem tissue, or wound tissue, can reconnect the stem zone above and below a similar girdle and that this wound tissue grows in a non-evenly distributed way (De Schepper et al., 2010). The downward flow of radiotracer also stopped immediately after the partial girdling; however, it already recovered after only 1 day (Figure 5B). This fast recovery indicates that the downward flow of assimilates probably found its way to the roots by changing its normal position toward undamaged phloem tissue.

Because the spatial pattern of phloem transport in the stem above the manipulated bark changed after partial girdling (or even complete girdling as soon as new transport tissue was available) translocation is clearly not absolutely determined by the vascular architecture of a tree stem (Preston, 1998). The pathway of assimilates delivered from a certain branch to the related sinks was markedly altered after partial girdling (Figure 5) possibly causing a branch to nourish another sink and changing its original sectoriality. Other studies (Gent, 1982; Aloni and Peterson, 1990; Preston, 1998) have observed a similar breakdown of sectoriality after manipulation of source-sink relations. These findings suggest that the barriers to lateral flow in the sieve tubes are not absolute and that sectoriality in the stem phloem is plastic (Preston, 1998; Orians et al., 2005).

Several hypotheses can explain this observed lateral transport and the altered movement of the assimilate flow following girdling. The first one is based on the leakage-retrieval mechanism happening in the transport phloem. According to this mechanism, a cycle of assimilate leakage out of the sieve tubes would exist, followed by retrieval back into the phloem (e.g., Minchin and Thorpe, 1987; Ayre et al., 2003; van Bel, 2003). Due to this leakage-retrieval mechanism, a certain sieve tube may retrieve substances leaking from another one at the same height in the tree. Such an exchange of content amongst sieve tubes is very likely in trees and woody plants where the winding nature of the phloem tissues results in periodic contact between sieve tube elements (Orians et al., 2005). Secondly, wounding could have induced lateral vascular interconnections between different vascular bundles, called anastomoses, which were not functioning under normal circumstances (Grusak and Lucas, 1984; Aloni and Peterson, 1990). In addition, in response to wounding, existing plasmodesmata can increase their size-exclusion-limit allowing larger openings between adjacent sieve elements (Schulz, 1995; Roberts and Oparka, 2003; Orians et al., 2005). Moreover, sap exchange between adjacent sieve tubes in a vascular bundle is possible, as lateral sieve areas exist in angiosperms (Walsh and Melaragno, 1976; Gattolin et al., 2008).

### Phloem speed can be independent of assimilate unloading

In the un-perturbed plant, phloem transport velocities of 8–10 mm min^−1^ (Figure 6) were at the lower range of the those observed in poplar trees with MRI (12–24 mm min^−1^, Windt et al., 2006) but were similar to velocities measured with ^11^C in stems of *Fraxinus excelsior* (2.5–10 mm min^−1^) and *Sorbus aucuparia* (5.4–9.5 mm min^−1^) for which measured transport velocities were dependent on both the position of the source leaves along the stem and the time of the season (Jahnke et al., 1998). In the absence of treatment, the velocity was more or less the same for the two labeled branches, and in both regions of the stem, suggesting that the velocity varied little within a cross-section, and was similar at the two observed heights. In contrast, although the loss was similar for both branches, it did vary with height. Clearly the carbon status of this stem varied with height, even without treatment. There was no externally obvious change in the stem anatomy over that region, such as a developing bud or wound. It may be that handling the plant was a cause, although that is unlikely since it was left un-moved for the 4 days between the similar results. Therefore it seems that the assimilate consumption, e.g., by phloem parenchyma for storage or by cambium cells for growth, differed along the axial transport pathway.

Whatever caused the axial difference in unloading, the difference of assimilate loss from the transport pathway could be expected to affect the axial osmotic gradient in the phloem. Thus if the transport followed a “simple” Münch mechanism (Münch, 1930), we would expect a roughly similar change in velocity to that in unloading (loss) assuming all other conditions (e.g., flow cross-sectional area) being equal along the transport path. Hence, it seems that plants tend to regulate their translocation in such a way that the translocation velocity is constant along the pathway. NMR measurements of several species showed a similar behavior over time: the phloem velocity was remarkably constant over the diel cycle (Peuke et al., 2001; Windt et al., 2006). A possible mechanism that can regulate phloem velocity is the leakage-retrieval mechanism (Gould et al., 2004; Thorpe et al., 2005; van Bel and Hafke, 2005). In the stem zone where the loss was higher, an active retrieval of older unlabeled carbon could have restored the sieve tube hydrostatic osmotic potential and hence pressure. In addition, other osmotica, such as potassium could play a role in regulating the phloem pressure (Thompson and Zwieniecki, 2005; Pritchard, 2007).

### Partial girdling changes the velocity and loss of assimilates

The decreased velocity of radiotracer flow from branch 1 after partial girdling (Figures 6A,C) can be explained by an increase in axial transport resistance caused by (enforced) lateral translocation. It is likely that the resistance of the lateral transport mechanism was higher than the resistance of the sieve tubes which were destroyed by the bark cutting. The temporary increase in velocity of flow from branch 2 after partial girdling (Figures 6A,C) could be due to a temporary higher sink demand, since the root system received less assimilates from all of the branches delivering assimilates via the girdled zone (including branch 1). The observed higher loss of radiotracer flow produced by branch 1 after partial girdling (Figures 6B,D) suggests that a smaller fraction of assimilates produced by this branch reached the lower stem and roots shortly after partial girdling. A similar compensatory increase in phloem flow of both water (Thorpe and Lang, 1983) and assimilate (Thorpe et al., 2011) has been observed when other sources are reduced, as by cold-girdling.

If the leakage-retrieval mechanism was responsible for the lateral transport then the loss for the pathway from branch 1 should have increased and remained high after partial girdling, because processes inducing the higher loss (e.g., increased energy demand for enhanced retrieval) would have not altered. In the case of an active change in the lateral translocation pathway (e.g., modification of plasmodesmata, activation of anastomoses), the amount of energy delivered by assimilates should temporarily increase to fuel the vascular changes. Hence, once these vascular changes are completed the required amount of energy delivered by branch 1 should reduce again. However, due to the large variability of the loss of branch 1 and the discrepancy of loss changes between the selected detectors at 3 days after girdling (Figures 6B,D), it is hard to distinguish between the suggested processes.

The modeled velocities appear to return to the initial velocity by 3 days after treatment (Figures 6A,C). A similar return to initial conditions (of phloem hydrostatic and osmotic pressure) was observed by Gould et al. (2004). The applied temperature treatment (Gould et al., 2004) increased the phloem pathway resistance; hence, it had a similar effect as the girdling treatment in our study. The restored velocity values in our study may therefore indicate that the increased pathway resistance was countered.

In conclusion, the PET system was successful in visualizing *in vivo* the ^11^C flow in the stems of young oak trees and in determining the vascular sectoriality of phloem transport in stems. The PET visualization supports the view that bark cuttings, which restricted normal phloem translocation, induced lateral translocation and altered sectoriality. Analysis of the temporal ^11^C-tracer profiles revealed that the fraction of transported assimilates lost along the transport pathway increased above the girdle, and that the velocity of the transported assimilates altered in response to girdling. In untreated trees, the transport velocity was constant along the transport pathway, while the loss could be variable.

### Conflict of interest statement

The authors declare that the research was conducted in the absence of any commercial or financial relationships that could be construed as a potential conflict of interest.
